# Management of the polyallergic patient with allergy immunotherapy: a practice-based approach

**DOI:** 10.1186/s13223-015-0109-6

**Published:** 2016-01-11

**Authors:** Pascal Demoly, Giovanni Passalacqua, Oliver Pfaar, Joaquin Sastre, Ulrich Wahn

**Affiliations:** Division of Allergy, Department of Pulmonology, Hôpital Arnaud de Villeneuve, University Hospital of Montpellier, Montpellier, France; Sorbonne Universités, UPMC Paris 06, UMR-S 1136, IPLESP, Equipe EPAR, Paris, France; Allergy and Respiratory Diseases, IRCCS San Martino-IST, University of Genoa, Genoa, Italy; Center for Rhinology and Allergology, Wiesbaden, Germany; Department of Otorhinolaryngology, Head and Neck Surgery, Medical Faculty Mannheim, Universitatsmedizin Mannheim, Heidelberg University, Mannheim, Germany; Allergy Division, Fundación Jimenez Díaz, Madrid, Spain; Department of Paediatric Pulmonology and Immunology, Charité Virchow-Klinikum, Humboldt University, Berlin, Germany

**Keywords:** Allergen immunotherapy, Allergy, Polyallergy, polysensitization, multi-allergen, single-allergen practice

## Abstract

**Background:**

The great majority (60–80 %) of patients consulting specialist physicians for allergic respiratory disease are polysensitized and thus may be potentially clinically polyallergic. However, management approaches to allergen immunotherapy (AIT) in polysensitized and polyallergic patients are not standardized.

**Methods:**

An international group of clinicians with in-depth expertise in AIT product development, clinical trials and clinical practice met to generate up-to-date, unambiguous, pragmatic guidance on AIT in polysensitized and polyallergic patients. The guidance was developed after reviewing (1) the current stance of regulatory bodies and learned societies, (2) the literature data on single- and multi-AIT and (3) the members’ confirmed clinical experience with polysensitized patients.

**Results:**

AIT is safe and effective in polysensitized and polyallergic patients, and should always be based on the identification of one or more clinically relevant allergens (based on the type and severity of symptoms, the duration of induced symptoms, the impact on quality of life and how difficult an allergen is to avoid). Single-AIT is recommended in polyallergic patients in whom one of the relevant allergens is nevertheless clearly responsible for the most intense and/or bothersome symptoms. Parallel 2-allergen immunotherapy or mixed 2-allergen immunotherapy is indicated in polyallergic patients in whom two causal relevant allergens have a marked clinical and QoL impact. In parallel 2-allergen immunotherapy (whether subcutaneous or sublingual), high-quality, standardized, single-allergen formulations must be administered with an interval of 30 min. Mixing of allergen extracts may be considered, as long as (1) the mixture is technically feasible, (2) the mixture is allowed from a regulatory standpoint, (3) the allergen doses are reduced in proportion to the number of components but are still at concentrations with demonstrated efficacy.

**Conclusions:**

Physicians can prescribe AIT (preferably with high-quality, standardized, single-allergen formulations) with confidence in polysensitized and polyallergic patients by focusing on clinical/QoL relevance and safety.

## Background

Allergic respiratory disease is a global health problem that seriously affects the sufferers’ daily lives [1–6]. Individuals with clinical symptoms of IgE-driven allergic respiratory disease will have specific IgE to disease triggering allergens as evidenced by skin prick tests (SPTs) or serum specific immunoglobulin E (ssIgE) assays. In surveys of the general population in Europe and the USA (performed with standard panels of allergens), polysensitization is generally more prevalent than monosensitization [7, 8]. In the first European Community Respiratory Health Survey, 12.8–25.3 % of the participants were polysensitized [7]. Similarly, the National Health and Nutrition Examination Surveys in the US found that 38.8 % of the participants were polysensitized [8].

Unsurprisingly, the great majority (60–80 %) of patients consulting allergists are polysensitized [9–12]. The prevalence of polysensitization increases with age [12–14], with 54 % in children under 11 years, 61.7 % in adolescents and 64.8 % in adults (p < 0.001) in the French ODISSEE study, for example [12]. Longitudinal birth cohort studies (such as the Multicenter Allergy Study in Germany [15], the Manchester Asthma and Allergy Study in the UK [16, 17] and the Barn Allergy Milieu Stockholm Epidemiology study in Sweden [18]) have shown that polysensitization is a risk factor for the subsequent development of allergic diseases in general and allergic asthma (AA) in particular. Polysensitization also impacts the clinical expression of the disease; the greater the number of sensitizations, the more severe the allergic disease [19]. Asthma is more likely to be associated with allergic rhinitis (AR) in polysensitized patients than in monosensitized patients [12].

However, a polysensitized patient does not necessarily have polyallergy, whereas a polyallergic patient is necessarily polysensitized. Polyallergy is defined as a documented, causal relationship between exposure to two or more specific, sensitizing allergens and the subsequent occurrence of relevant clinical symptoms of allergy (Table 1). Once polyallergy has been diagnosed, the physician’s next challenge is to decide (in collaboration with the patient) on a treatment strategy.

**Table 1:** Definitions

Term	Definition
Allergen sources	Allergen sources are allergens from the same homologous group (e.g. *Dermatophagoides pteronyssinus* and *Dermatophagoides farinae*)
Monosensitization	Sensitization (according to standardized SPTs or ssIgE assays) to only one of the allergens tested in the patient to date. A single “allergen” is defined in allergists’ terms, i.e. grass pollen, tree pollen, house dust mite, cat dander (even though extracts of these extracts contain tens, hundreds or even thousands of different polypeptides)
Polysensitization	Sensitization (according to standardized SPTs or ssIgE assays) to two or more allergens
Monoallergy	Clinically confirmed allergy to a single, sensitizing allergen (i.e. a causal relationship between exposure to the sensitizing allergen and clinical allergy symptoms)
Polyallergy	Clinically confirmed allergy to two or more sensitizing allergens (i.e. causal relationships between exposure to two or more sensitizing allergens and clinical allergy symptoms)
Homologous group	A group of allergens with (1) comparable physicochemical and biological properties of the source material, (2) cross-reactivity/structural homology of the allergens, (3) identical formulation of the finished product, and (4) identical production process of the allergen extract and of the finished product, as defined by the European Medicines Agency
Allergen mixture	A single formulation containing a mixture of several allergen sources (e.g. a grass pollen source mixed with a birch pollen source)
Single-allergen immunotherapy	Administration of an allergen immunotherapy formulation containing a single allergen source In the cases of sequential administration of two consecutive single-allergen immunotherapies.(e.g. 3 years of treatment with a house dust mite source, followed by 3 years of treatment with a grass pollen source) this does not constitute multi-allergen immunotherapy (see below)
Multi-allergen immunotherapy	Administration of different allergen sources Multi-allergen immunotherapy can be administered either in parallel (see below) or on a mixed formulation (see below)
Parallel multi-allergen immunotherapy	The separate administration of two or more single-allergen immunotherapy formulations in parallel during the same course of treatment
Mixed multi-allergen immunotherapy	The administration of an allergen mixture (i.e. a single formulation containing several allergen sources)

According to recent guidelines from the International Collaboration in Asthma, Allergy and Immunology [20], allergen immunotherapy (AIT) is indicated for the treatment of moderate-to-severe intermittent or persistent symptoms of AR—especially in those who do not respond well to pharmacotherapy. However, in the recently updated WAO position paper [6] on sublingual allergen immunotherapy (SLIT), failure of pharmacological treatment is not an essential prerequisite, and SLIT may be considered as an initial treatment for AR, in association with pharmacotherapy. We also consider that there are three additional indications for AIT: (a) the wish to avoid constant or long-term pharmacotherapy, (b) poor tolerability of symptomatic medications and (c) the wish to achieve a “cure” and possibly prevent disease progression (e.g. the development of new sensitizations and/or asthma) [21].

## Methods

Clinical practice in the diagnosis of respiratory allergy and its management with AIT vary from one country to another. When AIT is prescribed, some allergists tend to treat the polyallergic patient with a single-allergen formulation (using the most clinically relevant allergen), whereas others prefer to prescribe either a mixture of two or more allergen extracts or two or more separate allergens [22]. Several publications have sought (to a lesser or greater extent) to address the management of the polyallergic patient [11–13, 19, 23–27] and the principles governing production and quality issues when mixing allergens in AIT preparations [27] but do not provide comprehensive, consensual, practical guidance.

Hence, we have designed a focussed, practice-based approach to the management of polyallergic patients. The objective of the present document is to provide physicians with clear, up-to-date, unambiguous, pragmatic, clinically relevant guidance on their day-to-day practice. To do so, we constituted an international group of renowned clinicians with in-depth expertise in AIT product development, clinical trials and clinical practice. The group developed consensual, unambiguous, pragmatic guidance on AIT in polysensitized monoallergic or polyallergic patients on the basis of (1) the literature data, (2) learned societies’ and regulatory agencies’ stances on the formulation and clinical use of multiple allergen immunotherapy (multi-AIT, such as a formulation containing two or more allergen extracts), and (3) the members’ confirmed clinical experience with polysensitized patients. The aim is to give clear-cut answers to the most frequent questions raised by practitioners and patients. However, these recommendations are not intended to replace a clinician’s clinical judgement and must to be adapted to suit each individual patient.

### Treatment of the polyallergic patient: a high-priority topic

Before presenting our recommendations, we first consider (1) the current stance of regulatory bodies and learned societies regarding multi-AIT, (2) the clinical trial evidence in favour of multi-AIT and (3) evidence from observational studies conducted in physicians’ practices or in the general population.

#### What is the regulatory authorities’ current stance on the composition of AIT formulations?

The European Medicines Agency (EMA) has published general guidelines for manufacturers on the preparation and composition of allergen extracts and mixtures of extracts [28]. We were unable to find any guidance from other agencies around the world, including the US Food and Drug Administration. The EMA guidelines are based on the principle of homologous groups, which notably takes account of the physicochemical and biological characteristics of allergen extracts (Table 2). The EMA recommends that allergists should mix non-related allergens as little as possible and should not mix seasonal and perennial allergens or allergens with proteolytic activity (such as extracts of HDMs, moulds and insects) without justification. Mixing allergens clearly has an impact on pharmaceutical parameters (stability and dosing) and clinical effects (optimal dose and safety). The EMA’s “homologous group” principal requires (1) description of the source materials’ physicochemical and biological properties, (2) definition of the allergens’ cross-reactivity and structural homology, (3) preparation of identical formulations of the final product, and (4) a guarantee that the extract’s production process does not vary. The homologous groups generally correspond to taxonomic families. Within a given homologous group, allergen extracts will be very similar in terms of the composition, the source material’s physicochemical and biological properties, the allergens’ structural homology (and thus cross-reactivity) and the production process [28]. Further details on the rationale (based primarily on protein sequence data and cross-reactivity) for the six suggested homologous groups have been published (Table 2) [29]. The EMA document states that “*to a limited extent, data on quality, safety and efficacy can be extrapolated from the representative source to other members of the homologous group*”; for example, clinical data on birch allergen extracts can be extrapolated to other Betulaceae, such as the alder [28]. However, it is not possible to extrapolate efficacy results from one homologous group to another or from a homologous group to allergens that cannot be included in a homologous group; for example, clinical data on birch cannot be extrapolated to cypress. The EMA insists that the number of allergen extracts in a mixture should be kept to a minimum (regardless of the homology/cross-reactivity of the individual extracts) and that the number and relative proportions of the individual active substances must be justified. Mixtures containing allergens that do not belong to the same homologous group must always be justified [28].

**Table 2:** The homologous groups suggested by the EMA [28] and Lorenz et al. [29]

Homologous groups		No homologous groups	
Tree pollen			
1. Suggested homologous group: birch/fagales		Non-grouped species: justification for mixing required	
* Betula verrucosa* = B. pendula* = *B. alba*	European white birch	*Fagus sylvatica*	European beech
* Alnus glutinosa*	Alder	*Acer* sp.	Maple
* Carpinus betulus*	Hornbeam	*Platanus* sp.	Plane tree
* Corylus avellana*	Hazel	*Populus* sp.	Poplar
* Quercus alba*	Oak	*Robinia pseudoacacia*	False acacia, locust tree
* Castanea sativa*	Sweet chestnut	*Salix* sp.	Sallow/willow
		*Tilia* sp.	Lime
		*Ulmus* sp.	Elm
		*Cryptomeria japonica*	Japanese cedar
2. Suggested homologous group: *Oleaceae*			
* Olea europaea*	Olive		
* Fraxinus excelsior*	Ash		
* Ligustrum vulgare*	Privet		
* Syringa vulgaris*	Lilac		
3. Suggested homologous group: *Cupressaceae*			
* Juniperus* sp.	Juniper		
* Cupressus* sp.	Cypress		
Grass and cereal pollen			
4. Suggested homologous group: sweet grasses, the Poaceae (Gramineae) family, Pooideae subfamily		Non-grouped species: justification for mixing required	
* Anthoxanthum odoratum*	Sweet vernal grass	*Cynodon dactylon*	Bermuda grass
* Avena sativa*	Oat	*Cynosurus cristatus*	Dogstail
* Dactylis glomerata*	Orchard grass/cocksfoot		
* Festuca* sp.	Meadow fescue		
* Holcus lanatus*	Velvet grass/Yorkshire fog		
* Hordeum vulgare*	Barley		
* Lolium perenne*	Perennial ryegrass		
* Phleum pratense*	Timothy grass		
* Poa pratensis*	Kentucky bluegrass		
* Secale cereale*	Cultivated rye		
* Triticum aestivum*	Cultivated wheat		
Additional *Pooideae* grass species, with reservations:			
*Agropyron* sp.	Couch grass/crested wheatgrass		
* Agrostis* sp.	Bent grass		
* Alopecurus pratensis*	Meadow foxtail		
* Arrhenatherum elatius*	False oat		
* Bromus* sp.	Brome grass		
Weed pollen			
5. Suggested homologous group: weed pollen species		Non-grouped species: justification for mixing required	
* Ambrosia artemisiifolia*, *Ambrosia trifida*	Ragweed	*Plantago* sp.	Plantain
* Artemisia vulgaris*	Mugwort		
* Parietaria judaica*, *Parietaria officinalis*	Pellitory		
MITES			
6. Suggested homologous group: house dust mites of the *Dermatophagoides* genus		Non-grouped species: justification for mixing required	
*Dermatophagoides pteronyssinus*	European house dust mite	*Acarus siro*	Flour mite
* Dermatophagoides farinae*	American house dust mite	*Glycyphagus domesticus*	House mite
		*Lepidoglyphus destructor*	Storage mite
		*Thyreophagus entomophagus*	Flour mite
		*Tyrophagus putrescentiae*	Storage mite
Insect venoms		Non-grouped species: justification for mixing required	
No homologous groups		All species	
Allergen extracts derived from vertebrates (extracts such as animal epithelia, hair, dander)		Non-grouped species: justification for mixing required	
No homologous groups		*Canis familiaris*	Dog
		*Felis domesticus*	Cat
		*Cavia porcellus*	Guinea pig
		*Cricetus cricetus*	Hamster
		*Equus caballus*	Horse
		*Mus musculus*	Mouse
		*Oryctolagus cuniculus*	Rabbit
		*Rattus* sp.	Rat
Moulds		Non-grouped species: justification for mixing required	
		All species	
No homologous groups		In case of justification of grouping of mould species, special emphasis on similar stability is necessary	

#### What are the current guidelines from learned societies on the management of polyallergic patients?

Recommendations from the GA^2^LEN/EAACI [23] clearly state that the number of sensitizations itself is less important than the clinical relevance of each allergen. In fact, a personalised approach should be based on the identification of the clinically relevant allergen and should consider the type and severity of symptoms, the longest duration of induced symptoms over the year, the greatest impact on quality of life (QoL) and how difficult an allergen is to avoid. When considering the composition of AIT formulations, the GA^2^LEN/EAACI guidelines do not recommend mixtures [23]. The Allergic Rhinitis in Asthma (ARIA) guidelines have continuously put forward the same principles [2–4]. The US AIT practice parameters have moved in the same direction [30]; the third update emphasizes that it is important to treat the patients only with relevant allergens. None of these guidelines, however, gives pragmatic recommendations on how clinicians can identify and manage polyallergic patients in their daily practice.

#### What is the clinical trial evidence for the efficacy and safety of multi-AIT in polyallergic patients?

Few well-designed, double-blinded, placebo-controlled studies have evaluated treatment with multi-allergen formulations [24, 31–34]. Accordingly, most meta-analyses published to date have evaluated AIT formulations containing a single allergen or several cross-reactive allergens, and have urged caution with regard to multi-AIT. However, multi-AIT is common practice in the majority of allergists’ practices in the USA and in 20–40 % of the prescriptions in Europe [22]. This approach needs more supporting data from large clinical trials before it can be validated as a treatment option in polyallergic patients [23, 31]. Although some clinical studies of multi-AIT have clearly demonstrated efficacy, the thousands of different mixtures used worldwide have not been sufficiently investigated. A review by Nelson identified 13 studies (published between 1965 and 2007) in which two or more unrelated allergens were simultaneously administered as subcutaneous allergen immunotherapy (SCIT, 11 studies) or as SLIT (two studies) [32]. Nelson concluded that sublingual or subcutaneous administration of two simultaneous extracts was effective, on the basis of four studies reporting greater efficacy than placebo or much the same efficacy as single-AIT. Only seven of the trials were double-blind, placebo-controlled, randomized trials; it is obvious that most of these would not meet current standards for pivotal trials for regulatory approval.

#### What is the real-life evidence for the efficacy and safety of AIT in polyallergic patients?

“Real-life” clinical practice in the diagnosis of respiratory allergy and its management with AIT varies from one country to another. When AIT is prescribed, some allergists tend to treat the polyallergic patient with a single-allergen formulation (using the most clinically relevant allergen), whereas others prescribe either a mixture of two or more allergen extracts or two or more separate allergen extracts. Real-life observational and post-marketing studies show that AIT is safe and effective in polyallergic patients. Although very few observational surveys have been performed in allergists’ practices in Europe, the published data are very instructive. In a French study of 2434 polysensitized patients [35] reported that AIT was prescribed to 84.3 % of the patients. Of those who received AIT, 72.5 % were receiving a single formulation. When a single formulation was used, it was usually a single extract (in 86 % of cases) or, less frequently, a mixture of two allergen extracts (12.8 %) or three or more extracts (1.1 %). For patients receiving two AIT formulations, each was almost always a single-allergen extract (in 97 % of these cases). Furthermore, the results of two open, prospective, observational studies in Germany demonstrated that (1) polyallergic patients benefited as much from 300IR SLIT birch as monoallergic patients did and (2) in polyallergic patients treated with a 5-grass pollen extract, tolerability and symptom relief did not depend on the concomitant use of other allergen extracts [36, 37].

Ciprandi et al. prospectively evaluated a group of 87 adult patients (mean ± standard deviation age: 29.7 ± 10.8) with AR and/or mild-to-moderate AA [38]. The mean number of sensitizations per patient was 3.5, and the most frequent sensitizing allergens were grass pollen (64.4 %), house dust mites (HDMs) (46 %) and *Parietaria* pollen (36.8 %). Fifty-nine patients (67.8 %) were treated with single-allergen SLIT and 28 (32.2 %) were treated with 2-allergen SLIT. Importantly, there was no difference in the clinical outcomes (symptom severity, rhinitis classification and QoL) between these two treatment groups. Similar results were found in 51 polysensitized children (mean age: 11.8) with AR and/or mild-to-moderate AA [39]. One, two and three allergens were prescribed in 82, 8 and 6 % of cases, respectively (with missing data in 4 %). One year of SLIT was associated with significant reductions in ocular, nasal, and bronchial symptom scores (p < 0.01) and rescue medication use (p < 0.01), relative to pre-treatment values [39].

Hence, in surveys of real-life clinical practice, allergy specialists appear to consider that polysensitization per se does not influence the indications for AIT [12, 35, 40]. One should remind that the interpretation of adverse reactions maybe challenging: the culprit allergen is difficult to identify when a mixture is administered.

Although prescribing AIT in polysensitized patients (who may be monoallergic or polyallergic) is not a problem for trained clinicians with experience in allergy, the management approaches are not standardized and there is no clear-cut decision tree to assist clinicians in their provision of high-quality care. In general, the absence of clear guidelines and practice parameters has prompted physicians to shy away from prescribing AIT to polyallergy patients.

### A practice-based approach

We asked the group the following questions and moved forward by consensus:

1. How can be a polyallergic patient be identified?
2. When is AIT with a single allergen source indicated?
3. When AIT is with two allergen sources indicated (mixtures or two parallel course of AIT)?
4. When should two allergen sources be administered concomitantly?
5. How should two allergen sources be administered sequentially?
6. Can SLIT be combined with SCIT for 2-allergen immunotherapy?
7. When is AIT with three or more allergen sources indicated?
8. Are there specific issues to be considered when treating the most frequent polyallergic profiles?
9. Are there any other specific considerations?

#### How can be a polyallergic patient be identified?

Two main diagnostic methods are at our disposal: SPTs and ssIgE assays, both of which can only demonstrate the patient’s sensitization to an allergen source. These results must be cross-correlated with the clinician’s clinical interpretation, so as to identify the allergen(s) associated with a clinical and QoL impact (based on the GA^2^LEN recommendations) [23].

Although allergen challenges (i.e. a nasal challenge, a conjunctival challenge or exposure in a challenge chamber) can reproducibly demonstrate the clinical relevance of a given sensitization, they are difficult to perform [41]. Furthermore, the SPT wheal diameter and ssIgE titre are of limited value for identifying clinically relevant causal allergens at the patient level, although they are very useful at the population level [42]. In contrast, component resolved diagnosis (CRD) may help the physician to identify clinically relevant causal allergens and to distinguish genuine polysensitization (“co-sensitization”) from polysensitization due to cross-reactivity (“cross-sensitization”) (Table 1). It is now clear that molecular diagnosis can help to tailor the individual AIT [43–48], and it was recently shown that the levels of ssIgE against Par j 2 and Bet v 1 may distinguish between sensitization and allergy [49, 50]. Furthermore, it has been reported that ssIgE levels may predict the clinical response to AIT [51–53].

In particular, it is important to identify the allergen source(s) which most impact(s) QoL when allergies to two or more allergens from different homologous groups are diagnosed (e.g. grass pollen + HDM). Polyallergic patients will necessarily be polysensitized; the physician’s key task is to establish which of the sensitizing allergens are relevant with regard to the clinical symptoms of allergy. We consider that a patient’s clinical history alone is often (but not always) enough to identify the clinically relevant allergen in allergic respiratory disease, although it remains sufficient for an aetiological diagnosis in most cases. By way of an example, Crobach et al. reported that when considering a diagnosis of AR, the predictive value of the clinical history alone was 82–85 % for seasonal allergens and at least 77 % for perennial allergens [54]. These values increased to 97–99 % when both SPT and ssIgE data were available for a given patient.

Following identification of the most clinically relevant allergen, the physician’s next decision is how to treat the polyallergic patient.

#### When is AIT with a single allergen source indicated?

**Fig. 1:**
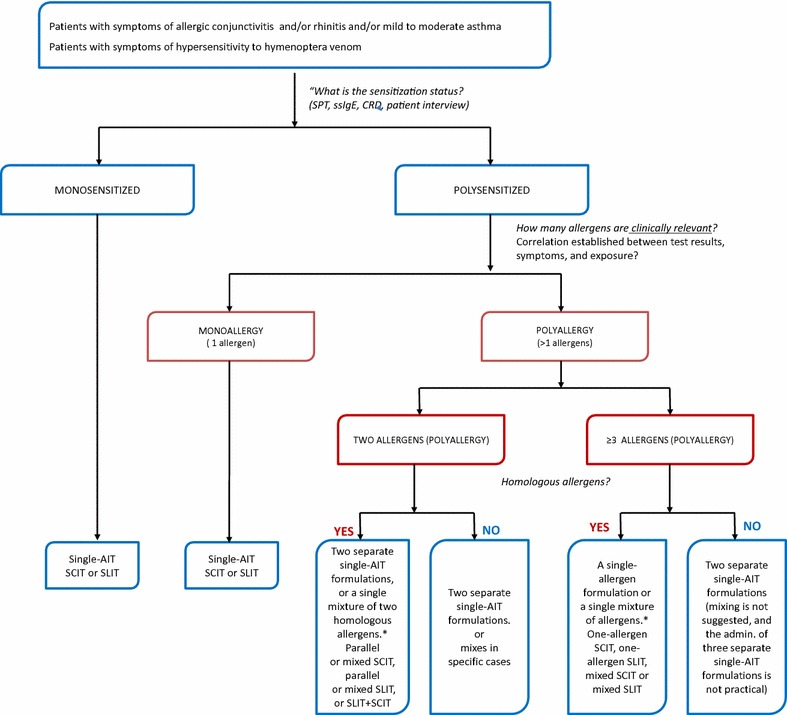
Suggested algorithm for AIT in polyallergic patients

Single-AIT is recommended in polyallergic patients in whom one of several relevant allergens is nevertheless clearly responsible for the most intense and/or bothersome symptoms. Again, the physician should identify this allergen on the basis of symptom intensity, impact on QoL, the duration of symptoms, and the ability to avoid allergens [23]. To facilitate the physician’s task, we have developed a treatment decision tree (Fig. 1). When selecting a treatment, patient preferences in relation to the administration route, adherence and cost, and the availability of high-quality AIT formulations must be taken into account.

Within a homologous group (such as *Dermatophagoides* species, Pooideae pollens or Betulaceae pollens), the use of a single course of AIT with a mixture of allergens that mimics natural exposure is recommended (e.g. a *Dermatophagoides pteronyssinus/Dermatophagoidesfarinae* mixture, a grass mixture or a birch/hazel/nut mixture). Patients are exposed and sensitized to allergen isoforms originating from two or more species in the group, and thus patients develop antibody and T cell responses to both cross-reactive (conserved) and non-cross-reactive (species-specific) epitopes. Therefore, a mixture of both species provides a broad spectrum of allergens and thus B and T cell epitopes for optimal reprogramming of the immune system [55].

Sublingual immunotherapy with one or several extracts was safe and effective in improving allergy-related outcomes [40, 56–61] (including a global asthma score, an asthma medication consumption score [59] and a health-related QoL score [56]) in both children [39] and adults aged 50 and over [58].

#### When is AIT with two allergen sources indicated (mixtures or two parallel courses of AIT)?

Parallel 2-allergen immunotherapy or mixed 2-allergen immunotherapy is indicated in polyallergic patients in whom two causal relevant allergens have a marked clinical and QoL impact. Our recommendations for the choice of AIT modality as a function of the clinically relevant allergen are summarized in Table 3. Treatment adherence and cost are both issues that may influence the physician’s decision to prescribe 2-allergen immunotherapy rather than a mixture. Should 2-allergen immunotherapy be indicated, it should preferably be administered as two standardized, single-AIT formulations in parallel (see below). Mixing of allergen extracts may be considered, as long as (1) the mixture is technically feasible (according to good manufacturing practice), (2) the mixture is allowed from a regulatory standpoint, (3) the various components are present at a concentration for which efficacy has been clearly demonstrated, and (4) the individual allergen doses in a mixture are adjusted (e.g. 1/2 of allergen source 1 and 1/2 of allergen source 2 in a two-allergen source mixture; 1/3 of allergen source 1, 1/3 of allergen source 2 and 1/3 of allergen source 3 in a three-allergen source mixture, etc). There is no scientific rationale for adjusting the mixing ratio mixture as a function of diagnostic test results, since the latter are not linked to clinical manifestations. However, mixing several allergen extracts is associated with a risk of (1) proteolytic degradation (as mentioned by the EMA) and (2) possible antigenic competition, due to saturation of the immune system’s allergen processing pathways at the administration site [24, 28, 62]. Although the latter subject requires further study, it has been reported that antigenic competition affects the immunogenicity and efficacy of injectable paediatric vaccines with antigens from six infectious pathogens [63].

**Table 3:**
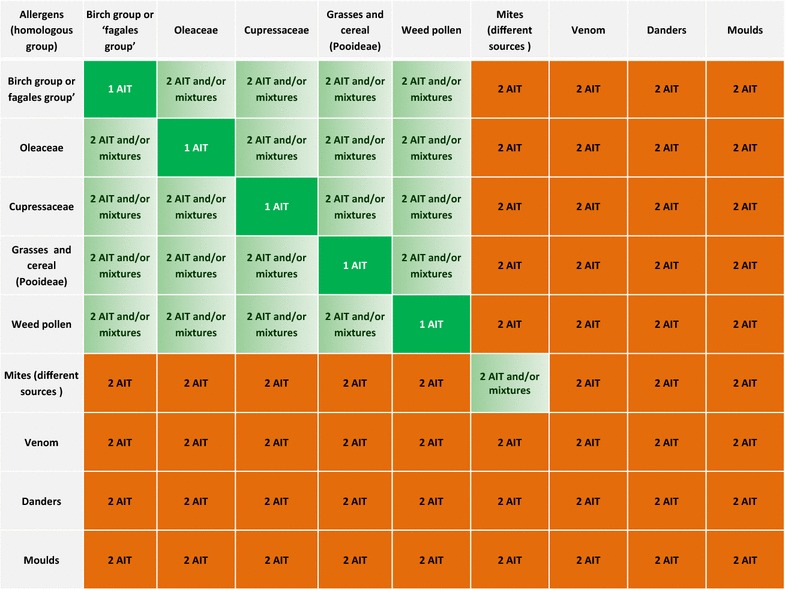
Recommendations if the patient is allergic to two clinically relevant allergens

Good adherence is a prerequisite for efficacy (regardless of the type of medication, and notably for AIT [64]), and so increasing the complexity of the treatment regimen will accentuate the importance of this parameter [65]. Likewise, two parallel courses of AIT will necessarily increase the cost of the treatments and associated procedures. Hence, cost and adherence issues may sometimes mean that two parallel courses of AIT are not indicated in a particular polyallergic patient; in some circumstances, a mixture might be an appropriate option.

#### How should two allergen sources be administered concomitantly?

Two-allergen immunotherapy can be administered as (1) a single mixture of two extracts (mixed multi-AIT, Table 1), with the standard ratio of each allergen source, or (2) two separate but simultaneous courses of one-allergen immunotherapy (parallel multi-AIT, Table 1). Use of separate AIT formulations is preferable when treating with two non-homologous allergens (as defined by the EMA). Most cases of polyallergy requiring the simultaneous administration of two clinically relevant allergens will involve non-homologous allergens (such as grass pollen + HDMs), rather than homologous allergens (such as olive pollen and ash pollen).

For SLIT (given its excellent safety profile), we recommend administering two separate SLIT formulations in the morning with an interval of 30 min (or one in the morning and one later on in the day). Although there may be an immune saturation effect at the oral mucosa [24, 62], this potential issue is avoided by ensuring an interval of 30 min between administrations of SLIT formulations. For SCIT, quasi-simultaneous injections at different sites/arms are commonly used by some experienced practitioners. However, the recommended 30-min observation period after each injection remains essential as a way of determining the responsibility of a particular allergen extract if adverse events occur.

There are few robust studies on the efficacy of mixed multi-AIT vs. parallel multi-AIT or on parallel vs. sequential administration [30]. Despite the absence of clinical trial results supporting the use of mixed allergens, there are no immunological reasons why a 2-allergen extract of homologous groups or two separate one-allergen extracts would lack efficacy.

#### When should two allergen sources be administered sequentially?

Allergen immunotherapy should be initiated first for the most clinically relevant allergen, then a subsequent course with the second more important allergen can be considered, at least 1 year after. Therefore we are speaking of 6 years at most.

However, lack of good adherence is a problem with both SCIT and SLIT when considered in terms of completing courses of therapy.

#### Can SLIT be combined with SCIT for 2-allergen immunotherapy?

A combination of SCIT and SLIT may be appropriate, subject to the patient’s preference and level of adherence, and the nature of the allergen source. For example, SCIT with a perennial allergen source and pre- and co-seasonal SLIT with a seasonal allergen will reduce the overall number of administrations. Both SLIT and SCIT are safe and effective when correctly prescribed and appropriately administered. By analogy with single-AIT (see above), the physician and the patient will decide together on the most appropriate administration route, as a function of personal preference and the availability of high-quality AIT formulations. However, if SLIT (or SCIT) is chosen because it is likely to be safe, effective and convenient for one course of treatment, it is likely to be so for a second course of treatment.

#### When is AIT with three or more allergen sources indicated?

We recommend focusing on the two most clinically relevant allergen sources. Hence, AIT with three or more allergen sources should only be considered in the very rare cases in which (1) all the allergens clearly cause severe symptoms and (2) a definitive molecular diagnosis (with CRD) is available prior to initiation of AIT. Even then, the physician should consider very carefully whether sequential treatments with a single-allergen formulation or several single-allergen formulations in parallel (together with on-demand symptomatic medications) will in fact be enough to provide the patient with adequate symptom relief. If AIT with three or more allergens is considered, its administration should follow the guidance given above for 2-allergen immunotherapy (i.e. administration at different times and body sites).

Prescriptions of AIT with three or more allergen sources are rare in European countries (and thus few data are available) but very common in the US [35]. In observational surveys in France, only 1.1 % of AIT prescriptions contained three or more allergen sources [36]. If a mixture of three or more allergen sources has to be used, individual doses must be adapted as described above, and this is likely to impact efficacy.

#### Are there specific issues to be considered when treating the most frequent polyallergic profiles?

Based on our experience in our respective countries, the following patient profiles will be most commonly concerned by the present guidance. In Europe, grass pollen + birch pollen is the most common polyallergic profile, followed by pollens + HDM. In a study in France, the most frequent polyallergy combinations were grass pollen-HDM (16.9 %), grass pollen-tree pollens (12.2 %), HDM-dander (10.6 %), HDM-dander-grass pollen (9 %), and HDM-tree pollen-grass pollens (8.1 %), albeit with regional variations. 76 % of the polyallergic patients presented with both seasonal and perennial allergies, 13 % suffered from perennial allergies only and 11 % suffered from seasonal allergies only [36].

The most common combinations of allergies in Germany are grass pollen + birch, grass pollen + HDMs, and tree pollen + HDMs; one should be aware that in Germany, mixing any other extract with a grass pollen, tree pollen or HDM extract would then mean that the resulting mixture becomes subject to the German Therapy Allergen Ordinance and thus would have to be approved by the regulatory authorities [66, 67]. In Italy, the most common combinations are grass pollen + HDMs, grass pollen + *Parietaria* pollen; HDMs + *Parietaria* pollen, grass pollen + birch pollen. In Spain, the most common combinations are grass pollen + olive pollen; grass pollen + *Cupressus* sp. pollen, *Salsola* pollen + grass pollen, *Cupressus* sp. pollen + olive pollen, grass pollen + *Parietaria* pollen, *Dermatophagoides* sp. + *Blomia tropicalis* and *Dermatophagoides* sp. + *Lepidoglyphus destructor.*

#### Are there any other specific considerations?

The physician must consider allergens such as the mite *Blomia tropicalis*, the subtropical Chloridoideae subfamily of grasses (e.g*. Cynodon dactylon*, Bermuda grass) and the Panicoideae subfamily of grasses (e.g. *Paspalum notatum*, Bahia grass)—especially for regional allergens or in (sub)tropical areas.

Ragweed pollen is a highly allergenic agent in some regions of Italy, France and eastern European countries.

*Alternaria* mould is also sometimes an issue (particularly in Spain), and so more studies of mould allergies are needed. With regard to animal dander, there is concern as to whether the doses of allergens in extracts are sufficiently high. Cockroaches and occupational allergens may be important in a few cases, although extracts are poorly standardized. In subtropical areas, the mite *Blomia tropicalis* has clear clinical relevance, and cross-reactivity with *Dermatophagoides* allergens is only partial. *Cynodon dactylon* may also be important and has limited cross-reactivity with the Pooideae [68]. These allergen sources deserve a thorough analysis of the literature and the provision of advice from experts in exposed areas.

## Conclusions

We recommend single-AIT in (1) polyallergic patients with a seasonal allergy to one allergen source and perennial allergy to another allergen, and (2) polyallergic patients in whom one of the several relevant allergens is clearly responsible for the most intense and/or bothersome symptoms. We recommended parallel or mixed 2-allergen immunotherapy only for patients in whom two allergens have similar, significant clinical and QoL impacts that overlap in time. When prescribed, 2-allergen immunotherapy should preferably consist of the separate administration of two high-quality, standardized, single-allergen formulations; this is highly preferable for non-homologous allergens. Simultaneous treatment with three or more allergens is rarely going to be clinically relevant. If a mixture has to be used, there is no reason for varying the ratios between the individual allergen sources (e.g. one should always use 1/2 of allergen source 1 and 1/2 of allergen source 2 in a two-allergen mixture; 1/3 of allergen source 1, 1/3 of allergen source 2 and 1/3 of allergen source 3 in a three-allergen mixture, etc), while ensuring that each of the components is still present at a concentration for which efficacy has been clearly demonstrated. All these preparations should used high-quality, evidence-supported and well standardized extracts as required by the World Allergy Organization [69].

## Abbreviations

AA: allergic asthma
AIT: allergen immunotherapy
AR: allergic rhinitis
CRD: component-resolved diagnosis
EAACI: European Academy of Allergy and Clinical Immunology
EMA: European Medicines Agency
GA^2^LEN: Global Allergy and Asthma European Network
IgE: immunoglobulin E
HDM: house dust mite
SCIT: subcutaneous allergen immunotherapy
SLIT: sublingual allergen immunotherapy
SPT: skin prick test
ssIgE: serum specific immunoglobulin E
